# Atrial Fibrillation Genetics Update: Toward Clinical Implementation

**DOI:** 10.3389/fcvm.2019.00127

**Published:** 2019-09-06

**Authors:** Silje Madeleine Kalstø, Joylene Elisabeth Siland, Michiel Rienstra, Ingrid E. Christophersen

**Affiliations:** ^1^Department of Medical Research, Bærum Hospital, Vestre Viken Hospital Trust, Rud, Norway; ^2^Department of Cardiology, University of Groningen, University Medical Center Groningen, Groningen, Netherlands; ^3^The Department of Medical Genetics, Oslo University Hospital, Oslo, Norway

**Keywords:** atrial fibrillation, genetics, genome-wide association studies (GWAS), heritability, precision medicine, personalized medicine, risk factors, whole genome sequencing

## Abstract

Atrial fibrillation (AF) is the most common heart rhythm disorder worldwide and may have serious cardiovascular health consequences. AF is associated with increased risk of stroke, dementia, heart failure, and death. There are several known robust, clinical risk predictors for AF, such as male sex, increasing age, and hypertension; however, during the last couple of decades, a substantive genetic component has also been established. Over the last 10 years, the discovery of novel AF-related genetic variants has accelerated, increasing our understanding of mechanisms behind AF. Current studies are focusing on mapping the polygenic structure of AF, improving risk prediction, therapeutic development, and patient-specific management. Nevertheless, it is still difficult for clinicians to interpret the role of genetics in AF prediction and management. Here, we provide an overview of relevant topics within the genetics of AF and attempt to provide some guidance on how to interpret genetic advances and their implementation into clinical decision-making.

## Introduction

Atrial fibrillation (AF) is the most common sustained heart rhythm disorder and is estimated to affect more than 30 million individuals worldwide (1). The lifetime risk of individuals older than 55 years of age is 37%, and AF is associated with an increased risk of stroke, dementia, heart failure, and death (2).

Many studies have investigated the risk factors for AF to improve prevention of the arrhythmia. The most important risk factors for AF are increasing age, male sex, hypertension, diabetes, obesity, myocardial infarction, heart failure, and stroke (3, 4); aggregation of risk factors further increases the risk for AF. However, the AF population is heterogeneous, and some individuals develop AF with no apparent risk factors. Despite all efforts, it remains difficult to predict AF based solely on clinical risk factors.

Ever since familial clustering of AF was demonstrated and the heritability of AF was established, large efforts have been made to identify the genetic causes of AF (2, 5, 6). With the advances of genetic sequencing and bioinformatic analyses, the field of genetic research has turned from candidate gene sequencing approaches to large-scale genome-wide analyses. Today, more than a hundred genetic loci have been associated with AF.

For clinicians, it remains difficult to interpret the genetic advances in the context of clinical risk factors, and translation of genetic discoveries to clinical decision-making remains unresolved. Here, we aim to provide clinicians with an overview of the relevant literature on the genetics of common AF using four directions earlier described (7): genetic mapping of AF, AF risk prediction, therapeutic development, and patient-specific management. We provide some guidance on the consequences of large-scale genetic studies for AF and whether genetic information is ready to be implemented into clinical practice—alternatively, what is needed to get there.

## Familial Clustering of AF

One of the first descriptions of familial aggregation of AF was published in 1943, describing three brothers diagnosed with AF who had experienced irregular heart rate since childhood (8). More recently, several studies have described familial clustering of AF in larger populations (5, 6, 9–12). In 2004, Fox et al. showed that one-third of the individuals diagnosed with AF in the Framingham Heart Study had at least one parent also diagnosed with AF, providing evidence that parental AF increased the risk of AF in offspring in the general population (5). Short after this publication, the Icelandic nation-wide genealogic community-based data were used in an analysis of more than 5,000 individuals. An important finding was that the AF risk ratio declined exponentially when the proportion of identically shared alleles by descent diminished in relatives, confirming the importance of genetics in relation to AF risk. Similarly, a Danish twin study showed that having a co-twin with AF increased an individual's risk of AF and that the risk was doubled in monozygotic twins, compared to dizygotic twins (6). In 2010, the Framingham Heart Study confirmed that familial AF, particularly when premature in onset, slightly improved prediction of new-onset AF beyond clinical AF risk factors (13). Moreover, the risk of AF increased with an increasing number of affected first-degree relatives, and having an affected sibling conferred a similar magnitude of risk as parental AF. Together, the observations suggest that familial clustering of AF may be genetically caused.

## Genetic Mapping of AF

### Segregation Analyses and Candidate Gene Sequencing

Historically, segregation analyses and candidate gene sequencing were used to identify genotype–phenotype correlations, involving families or populations with high prevalence of AF. Segregation analysis is used to identify genetic markers co-segregating with the AF phenotype, utilizing the linkage phenomenon; because of genetic recombination, genetic markers that are located in close proximity are more likely to co-segregate through pedigrees than genetic markers located far apart. In 2003, a gain-of-function mutation in KCNQ1 was identified through segregation analysis, making it the first AF-associated gene (14). Candidate gene sequencing involves sequencing of candidate genes, based on our knowledge of their biological function, and thus an *a priori* hypothesis that increased or decreased function of the resulting proteins may lead to AF. Although many genetic variants, largely related to ion channels, have been associated with AF using segregation analysis and candidate gene sequencing, there are several limitations to these methods. The limited sample size in most of these studies and the low frequency of the genetic variants discovered have made them underpowered. The recent introduction of large reference databases of exomes and genomes has shown that many genetic variants proposed to be related to AF and other cardiac arrhythmia disorders through candidate gene sequencing are present at a much higher frequency in the population than what we would expect of a disease-causing variant (15–18).

### Genome-Wide Association Studies on AF

**Figure 1:**
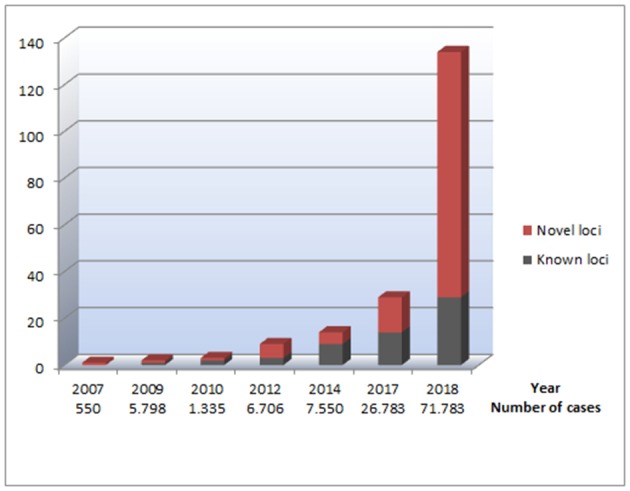
An overview of genetic loci identified through GWAS performed for AF. The figure illustrates the number of identified loci for AF, based on the data listed in Table 1. The x axis represents which years the different GWAS for AF was performed. Under each year, the total number of cases included in all GWAS for AF the current year is listed, illustrating the relationship between the increasing sample sizes and the increasing number of AF-associated loci identified. The Y axes represent the number of genetic loci associated with AF. The red parts of the columns represent genetic loci that have not previously been reported in relation to AF. The black parts of the columns represent previously reported genetic loci associated with AF, making the total of each column reflect the total number of AF-associated loci at a given time.

The new millennium brought along the genome-wide association study (GWAS) approach, which was novel in its unbiased nature and its focus on common rather than rare genetic variants. One of the first GWAS was performed in 2002 by Ozaki et al. who identified a candidate locus associated with myocardial infarction (19). This achievement initiated an accelerated search for disease-associated loci through GWAS. The first large GWAS for AF, including 550 AF cases, was performed on Iceland in 2007 (20), identifying a genetic locus associated with AF on chromosome 4q25. The most significant variant was located in an intergenic region, with the nearest gene, *PITX2*, 150 kilobases (kb) away. Pitx2c is a homeodomain transcription factor involved in embryonic development, essential in the formation of the atria, the sinus node, and left-right heart asymmetry (20, 21). Figure 1 shows an overview of GWAS performed for AF to date. The association between the 4q25 locus and AF was replicated in two independent studies in 2009 (22, 23), and since then, *PITX2* has continued to be the most significant genetic locus in AF GWAS. Gudbjartsson et al. expanded their GWAS to include 2,385 individuals with AF, and identified the *ZFHX3* locus in 2009 (24). In the same period, Benjamin et al. showed a similar association between the *ZFHX3* locus and AF in a European ancestry population (25). The *ZFHX3* gene encodes a transcription factor enriched in cardiac tissue (21, 25), and there is evidence showing that interaction between ZFHX3 and Pitx2c or TBX5, both transcription factors active in embryogenic cardiogenesis, may increase the risk of AF significantly (26, 27).

In 2010, the *KCNN3* gene, encoding a calcium-gated potassium channel involved in atrial repolarization, was identified as the third AF-associated locus in a lone AF population by Ellinor et al. Two years later, the first GWAS meta-analysis from the international Atrial Fibrillation Genetics (AFGen) Consortium, including ~6,500 AF cases, revealed six new genetic loci for AF (28). The most significant novel association was found for the *PRRX1* locus, which encodes a transcription factor highly expressed in the developing heart. Further studies on *PRRX1* have shown that loss of *PRRX1* expression results in shortening of the atrial action potential duration and may, thus, promote AF (29).

From 2007 to 2017, there was a steady increase in the identification of novel GWAS loci for AF, and a total of 14 new loci were identified in this period (20, 24, 25, 28, 30, 31) (Table 1).

**Table 1:** Overview of GWAS for AF.

**Year**	**Cases (*n*)**	**Referents (*n*)**	**Ancestry**	N new loci^*^	Total *N* loci^**^	**Authors**	**References**
2007	550	4,476	European (Iceland)	1 *(4q25 (PITX2))*	1	Gudbjartsson et al.	(20)
2009	2,385	33,752	Mostly European ancestry, ~11% Chinese	1 *(ZFHX3)*	2	Gudbjartsson et al.	(24)
2009	3,413	37,105	European	0 *(ZFHX3)*	2	Benjamin et al.	(25)
2010	1,335	12,844	European	1 *(KCNN3)*	3	Ellinor et al.	(30)
2012	6,707	52,426	European	6 *(PRRX1,CAV1,C9orf3, SYNPO2L (MYOZ1), SYNE2, HCN4)*	9	Ellinor et al.	(28)
2014	7,550	55,776	European	5 *(NEURL, CAND2, GJA1, TBX5, CUX2)*	14	Sinner et al.	(31)
2017	17,931	115,142	European, Asian, and African-American	12 *(METTL11B–KIFAP3, ANXA4–GMCL1, CEP68, TTN/TTN-AS1, KCNN2 KLHL3–WNT8A–FAM13B, SLC35F1–PLN, ASAH1–PCM1 SH3PXD2A, KCNJ5, SCN10A, SOX5)*	26	Christophersen et al.	(32)
2017	8,180	28,612	Asian (Japanese)	6 *(KCND3, PPFIA4, SLC1A4–CEP68, HAND2, NEBL and SH3PXD2A)*	31	Low et al.	(33)
2017	672	3,700	Asian (Korea)	0 *PPFIA4, HAND2*	31	Lee et al.	(34)
2018	6,337	61,607	European	1 *1p32*	32	Nielsen et al.	(35)
2018	65,446	≈500,000	Combined 84.2% European, 12.5% Japanese, 2% African American, 1.3% Brazilian and Hispanic populations	70	102	Roselli et al.	(36)
2018	60,000	970,216	European	≈10	≈134	Nielsen et al.	(37)

*The table gives an overview of GWAS for AF from the first locus reported in 2007 until 2018*.

* *Number of genetic loci that have not previously been reported in relation to AF*.

** *Total number of genetic loci associated with AF, summed up by previously reported loci in addition to new loci of the current year. The data from the table are illustrated in Figure 1*.

In 2017, Christophersen et al. identified 12 new loci associated with AF in a multiancestry GWAS meta-analysis from the AFGen Consortium, nearly doubling the number of AF risk loci. Their study included European, Asian, and African-American ancestry groups (32). An intriguing finding was the *TTN* locus, which encodes titin, the largest protein in the human body and an essential building block in the sarcomeres of striated muscle tissue. It is highly expressed in the human heart, in both atria and ventricles. Hence, titin dysfunction can alter the cardiomyocyte structure (38). A second biologically interesting discovery was the gene *KCNN2*, encoding the calcium-dependent potassium channel subunit SK2. SK2 forms a complex with SK3, which is encoded by the gene *KCNN3* previously associated with AF in an early AF GWAS, as described above (30). *SK2* blockers are under development as a new potential treatment against AF (39–42). GWAS in Korean and Japanese populations the same year identified six loci, of which the loci *KCND3, PPFIA4, HAND2*, and *NEBL* were specific to East-Asian AF patients (33, 34).

In 2018, a Norwegian GWAS including 6,337 cases and 61,607 referents (35) identified one novel risk locus on chromosome 1p32, with *DMRTA* and *CDKN2C* as suggested functional genes, potentially involved in structural remodeling of cardiac tissue as a substrate for AF.

Later in 2018, there was a major leap in the progression of AF GWAS when a large GWAS meta-analysis from the AFGen Consortium revealed 97 AF risk loci, of which 92 represented common variants and 70 were novel findings (36). The study included over half a million individuals of combined ancestry and approximately 65,000 AF cases. Another GWAS meta-analysis of the same magnitude was published the same year, including ~1 million individuals, of which ~60,000 were AF cases (37), identifying 111 AF-associated loci. A preliminary meta-analysis combining non-overlapping participants from these two largest GWAS performed, including ~93,000 AF cases, resulted in ~134 genetic loci associated with AF (36).

The new genetic discoveries provide optimism for future novel treatment options for AF, as many of the candidate genes identified may provide potential novel drug targets. Roselli et al. points out the *SCN5A* gene, encoding the most important sodium channel in heart tissue, and *KCNH2* encoding a subunit of the potassium channel complex, as primary targets for antiarrhythmic channel blockers or inhibitors already in use (36). Of the 151 candidate genes identified, Nielsen et al. found potential drug interactions for 31 genes, describing a total of 475 potential drugs (37). Of the potential drugs identified, 78 are already known in relation to treatment for arrhythmias. For some of the drug target interactions, clinical trials may be in sight, and as mentioned above, the *SK2* blocker seems to be a promising new treatment for AF.

It has been proven possible to carry out large AF GWAS meta-analyses in an order of magnitude that provides the statistical power needed to establish true genetic associations, through extensive international collaboration. So are we there yet, or do we need even bigger and better GWAS?

In order to approach an answer to this question, we can look to large GWAS made for other common complex diseases, such as major depressive disorder or schizophrenia. In 2018, Nishino et al. predicted the number of cases needed to detect significant genetic variants for these two psychiatric diseases (43). The numbers of cases needed to detect 1, 10, and 100 genetic variants by GWAS were calculated to be 7,000, 18,000, and 51,000 cases for schizophrenia, and 34,000, 61,000, and 118,000 cases for major depressive order, respectively. The authors pointed out that the number of genetic variants associated to major depressive disorder would rapidly increase, when 50,000 cases or more were included. In comparison, the largest AF GWAS thus far included a similar number of cases.

However, the power of a GWAS depends not only on the sample size, but equally important are the genetic coverage (genotyping chip and imputation panel density), the frequency of the genetic variants, the ancestry group, and the effect size (44–48). Nelson et al. have estimated the power of GWAS across a range of genotyping chips, ancestry groups, minor allele frequencies, and effect sizes, suggesting that we are close to having identified the most relevant common variants for AF in European ancestry groups. The pursuit of new common variants for AF will likely be more successful if we focus on non-European ancestry groups and on low-frequency variants with large effect size (46). Moreover, as discussed below, heritability estimates show that there is still a large proportion of missing heritability, which may partially be caused by unidentified common genetic variants.

### Heritability of AF

The large number of common genetic variants now identified for AF has enabled estimation of the heritability of AF, using genetic data. In 2017, Weng et al. showed that genetic variation accounts for 22.1% of the total variance for AF (2), of which the 25 AF loci that were known at the time accounted for only 25%. The fraction of the heritability of AF explained by common genetic variants have increased to 42% based on the results of the largest GWAS thus far (36). As evident from the estimates of explained heritability, there is still a substantial proportion of “missing heritability.” Where the missing heritability is buried is debated. First, common genetic variants identified through GWAS are mostly located in intergenic or intronic regulatory regions. They may participate in disease development through regulatory mechanisms, thereby affecting the expression of multiple genes located in proximity and possibly further away. For example, the co-regulatory network involving *TBX5* and *PITX2* influences atrial development (36, 49). Gene–gene interactions can play a significant role in regulating cellular behavior and biological events. Two variants with small individual effects may have large interaction effects. Lin et al. showed that two genetic variants, rs7164883 at the *HCN4* locus and rs4980345 at the *SLC28A1* locus, were highly significantly associated with AF in a European ancestry discovery cohort from 15 studies. Moreover, eight additional gene–gene interactions were marginally significant, albeit unreplicated thus far (50).

Second, gene–environment interactions may contribute to the missing heritability. Some cross-trait associations have been found between genetic variants associated with AF risk factors, such as height, body mass index (BMI), and hypertension, and AF risk variants (36). These pleiotropic effects may also contribute to missing heritability. Last, unidentified common and rare genetic variants, including copy number variation, may partially explain the missing heritability of AF (51). The fast-developing high-throughput sequencing technology may play an important role in uncovering missing heritability for AF.

### High-Throughput Sequencing in AF

**Figure 2:**
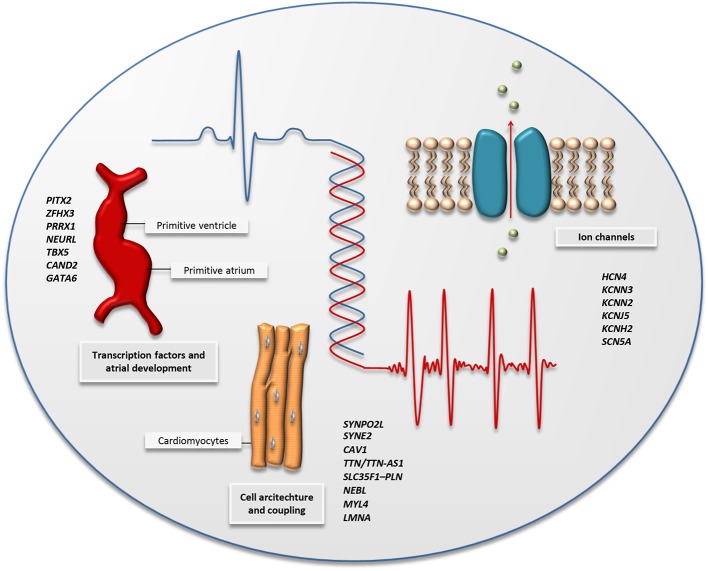
The figure illustrates the main biological pathways implicated by AF-associated variants identified by GWAS and high-throughput sequencing; cardiac transcription factors and embryonic cardiogenesis, the architecture of the cardiac cells, and ion channel function. A selection of genes associated with AF through GWAS and high-throughput sequencing is listed for each pathway and can also be found in Tables 1, 2.

During the last decades, genetic sequencing technology has evolved rapidly, and with that, the price for sequencing a whole genome has dropped dramatically (52). High-throughput sequencing describes a variety of techniques, including whole-exome and -genome sequencing, that enable high-resolution genetic analysis of all coding and non-coding regions of our genome. The advantage of high-throughput sequencing is the ability to analyze genetic variants with lower frequencies, which are more likely to have large effects on disease risk, as opposed to GWAS, which is mainly suitable for analysis of common variants that confer small effects on disease risk. Table 2 gives an overview of the main published high-throughput sequencing studies of AF. Figure 2 shows the main biological pathways implicated in AF pathophysiology by GWAS and high-throughput sequencing studies.

**Table 2:** Overview of high-throughput sequencing studies for AF, divided in family- and population-based studies.

	**Year**	**Cases (*n*)**	**Referents (*n*)**	**Study design**	**Genetic loci**	**Biological implication**	**Authors**	**References**
Family-based studies	2014	18 (six families)	0	Whole exome	39 very rare potentially pathogenic variants	Unknown	Weeke et al.	(53)
	2014	20 trios + AF: 200	200	Targeted exome sequencing of AF-related genes + Sanger	*PITX2 SYNE2 ZFHX3 KCNN3*	Cardiac development Cardiac contractility/structural Cardiac development Cardiac electrical function/ion channel subunits	Tsai et al.	(54)
	2016	AF: 5 family members Healthy relative: 2	100	Whole exome	*MYL4*	Cardiac contractility/structural	Orr et al.	(55)
	2016	AF: 3 family members Healthy relatives: 1	524	Whole exome + Sanger	*LMNA*	Cardiac contractility/structural	Zhao et al.	(56)
	2017	AF: 4 family members Healthy relatives: 4		Gene panel testing+ Sanger	*SCN5A*	Cardiac electrical function/ion channel subunits	Lieve et al.	(57)
	2017	AF: 3 family members + AF early-onset: 546	6,500	Whole exome	*GATA6*	Cardiac development	Tucker et al.	(58)
	2018	AF: 77 + AF early-onset: 399	663	Whole exome	*TTN*	Cardiac contractility/ structural	Ahlberg et al.	(59)
Population-based studies	2015	WGS: 2,636 Homozygous carriers of *MYL4* c.234delC variant: 8		Whole genome	*MYL4*	Cardiac contractility/ structural	Gudbjartsson et al.	(60)
	2016	AF: 1,743	9,423	Whole- exome	No significant associations		Lubitz et al.	(61)
	2018	AF: 2,781	4,959	Whole genome	*TTN*	Cardiac contractility/ structural	Choi et al.	(62)

### High-Throughput Sequencing in Family Studies

One of the first reports of high-throughput sequencing in AF was performed in 2014, where 39 very rare variants [minor allele frequency (MAF) <0.04%] were identified through whole-exome sequencing in six families with aggregation of AF (53). The potentially pathogenic variants, with a range of 7 to 15 shared variants per family, underscored the complexity of the genetics of AF and suggested that non-coding regions may be more important in the search for causal genetic variants. However, the lack of sequencing of healthy family members leaves us uncertain whether the identified genetic variants truly segregate with the disease. The same year, five novel rare variants were identified performing high-throughput sequencing of nine AF-associated genes in 20 parent–offspring trios (54). One of the variants was located in the 5' untranslated region of the *PITX2* gene, downregulating expression of Pitx2c in atrial myocytes. The remaining variants were exonic nonsense mutations in the genes *SYNE2, ZFXH3*, and *KCNN3*, and thus represent each of the three main pathological pathways for AF development suggested by prior GWASs (Figure 2). Whereas, mechanisms for AF development previously have been focused at cardiac electrical function, there are several genetic findings that now also point to structural changes in the atrium as a cause of the arrhythmia. Orr et al. highlighted one such association describing a family with aggregation of early-onset AF, cardiac conduction disease, and atrial myopathy (55). Through whole-exome sequencing, they identified a novel genetic variant in *MYL4* shared by all five affected relatives. *MYL4* is expressed in adult atria and embryonic muscle tissue, and it has been shown that loss-of-function variants in *MYL4* can lead to atrial cardiomyopathy (63). Another genetic variant related to structural changes in the heart, a non-sense mutation in the *LMNA* gene (c.G1494A, p.Trp498Ter), was identified through whole-exome sequencing and Sanger sequencing in a four-generation AF family from northern China (56). *LMNA* encodes the nuclear membrane proteins Lamin A and Lamin C, and pathological variants are known to cause a broad variety of inherited diseases referred to as laminopathies, including myopathies in skeletal muscle with a dystrophy-like picture, cardiomyopathy, and conduction system disease (64–66).

In 2017, Lieve et al. performed a gene panel testing complemented with Sanger in a two-generation Dutch family, with a phenotype presenting with ventricular arrhythmias and early-onset AF (57). They found a gain-of-function variant in *SCN5A*, which encodes the alpha subunit of the main cardiac sodium channel, and with this, drew attention back to cardiac electrical function as a biological pathway for AF. The functional effect of the variant seemed to be increased channel availability and current duration. *SCN5A* has previously been shown to be one of the most important genes for overall atrial conduction (32). The same year, Tucker et al. identified a gain-of-function variant in *GATA6* through whole-exome sequencing in two families with early-onset AF; one of the families also displayed atrioventricular septal defects (58). *GATA6* encodes a cardiac transcription factor important to cardiac morphogenesis, and loss-of-function variants have previously been reported to be associated with congenital cardiac defects (67, 68). In addition, loss-of-function variants in *GATA6* have been associated with lone AF through candidate gene studies (69, 70).

As described, the gene *TTN* has been associated with AF in two independent GWAS meta-analyses (32, 35), and results from recent high-throughput sequencing studies support this association (59, 62). Ahlberg et al. performed whole-exome sequencing in 24 families with aggregation of AF (59) and discovered that the frequency of titin-truncating variants (*TTNtv*) in cardiac transcripts was significantly higher in the individuals with AF compared to an unaffected referent group (16.7 vs. 0.5%, *P* = 1.76 × 10^−6^). The association was replicated in 399 individuals with early-onset AF (AF before age 40), showing an increased frequency of *TTNtv* in cases compared to referents (odds ratio [OR] = 36.8, 95%, confidence interval [CI] = 5.0–4692.5, *P* = 4.13 × 10^−6^). They further examined the effect of *TTNtv* on atrial development in a zebrafish model, which revealed disorganized sarcomeres and atrial fibrosis in adult heterozygous fish, and electrocardiogram (ECG) analysis showed prolongation of the PR interval, which is a known risk factor for AF (71).

### High-Throughput Sequencing in Population-Based Studies

Large-scale high-throughput sequencing is merely in the initial phase when it comes to AF in the general population. In 2015, Gudbjartsson et al. performed large-scale whole-genome sequencing in 2,636 individuals on Iceland (60). They found a recessive frameshift mutation in *MYL4* (c.234delC, MAF = 0.65%), increasing the risk for early-onset AF by 110-fold (recessive OR = 110.3, *P* = 5.2 × 10^−10^). Eight homozygous carriers of the *MYL4* variant were identified. All eight carriers had been diagnosed with early-onset AF. The need for large sample sizes in order to have sufficient power to detect significant associations in high-throughput sequencing studies was demonstrated by Lubitz et al. who performed whole-exome sequencing in 1,734 individuals with AF and 9,423 referents, with no significant associations detected (61). Recently, Choi et al. reported results from whole-genome sequencing in almost 2,781 individuals with early-onset AF and 4,959 referents of European ancestry (62). They found an association between a rare (MAF = 0.1%) loss-of-function variant in *TTN* and early-onset AF and showed that the probability of being a carrier of the variant increased conversely with age at AF onset. As previously described, *TTN* encodes the large protein titin, which forms a crucial component in the sarcomeres of muscle tissue. An association between *TTN* variants and dilated cardiomyopathy (DCM) is well-established (72, 73), and patients with familial or early-onset DCM are routinely screened for variants in the gene (74). The recent discoveries indicate that *TTNtv* variants may contribute to AF without the presence of DCM (59, 62).

The population-based studies describing a role for *TTN* and *MYL4* in the genetic background for AF are in line with the findings from the family-based studies by Orr and Ahlberg, described above. The results suggest that a proportion of AF-related genes or genetic regions may be involved in structural cardiac abnormalities, in addition to the well-known electrical abnormalities identified for several ion channel genes, and support the hypothesis that some types of AF may be considered to be atrial cardiomyopathies (75). The obvious fact that AF, as a heterogeneous disease, displays different causal mechanisms is important for differentiating treatment. In the future, we may routinely be identifying underlying causes of AF through genetic testing, before choice of treatment.

### Mendelian Randomization Studies—Causal Effects of Genetic Variants

In a polygenic and heterogeneous disease, such as AF, it is difficult to prove causality. Causality can only be established when confounding factors are eliminated in the analyses. Residual confounding, reverse causation, and bias should be taken into consideration in any analyses of observational data (76, 77). Mendelian randomization is a statistical method that is currently used as a powerful control for reverse causation and confounding. In Mendelian randomization, genetic variants with known association to the exposure are used as instrumental variables to examine the causal effect of the exposure on the disease. The use of genetic variants of AF-related risk factors may clarify the complexity between AF and the environmental factors that interact with AF. A causal relation between BMI and incident AF has been suggested by observational data (4, 78), until recently confirmed by Chatterjee et al. using Mendelian randomization (79), supporting prevention of obesity as a therapeutic target to reduce incident AF. However, BMI covers weight and height but does not define the components of body mass, e.g., distinguish between fat mass and muscle mass. In an attempt to understand how body mass plays a causal role in AF, Tikkanen et al. evaluated the relation between fat mass and fat-free mass, and incident AF, using multivariate Mendelian randomization (80). During a follow-up period of 6.1 years, 10,852 incident AF cases occurred in the UK biobank population of 502,619 individuals (54% women, mean age = 56.5 ± 8.1 years). The multivariate Mendelian randomization showed that the effects of fat mass and fat-free mass were independently of each other associated with incident AF. Moreover, fat mass showed stronger causal associations in women, whereas fat-free mass displayed a similar association across sexes. Reductions in fat mass and fat-free mass likely follow different biological pathways, and both reduce AF risk independently.

Another anthropometric trait, height, is a well-known, independent risk factor for AF (81). More than 400 genetic loci have been associated with height (82–86), of which selected variants have also been associated with AF (87). Taller individuals have larger atria and more frequent premature atrial contractions. These are both strong predictors for AF and can influence the cardiac conduction system, reflected in PR interval and QRS duration on ECG (88–92). Kofler et al. investigated 2,149 individuals [54% women, median age = 37 (30–40) years], with a median height of 1.71 m, of the Genetic and Phenotypic Determinants of Blood Pressure and other Cardiovascular Risk Factors study (93). The Mendelian randomization showed a significant association between height and both PR interval and QRS duration. The authors propose that genetically determined body size has impact on the cardiac conduction system and thus may lead to increased risk of AF. However, the association may not be generalizable to all populations. The genetic variants associated with height discovered in data of the GIANT consortium and replication studies have been suggested to be biased by population stratification (94–97). The suggested polygenic adaptation of height, supported by average polygenic scores, increased from south to north across Europe, but in such steep line that is inconsistent with a neutral model evolution. Recently, new analyses were performed in the UK Biobank, in which the signals of selection were absent. Berg et al. highlights the challenge that comes with correcting for population stratification in GWAS of polygenic traits and distinguishing differences in polygenic scores between populations.

Hyperthyroidism increases the risk of AF, and hypothyroidism is associated with a reduced risk of AF. The increased risk or AF persists despite treatment against hyperthyroidism (98, 99). In a Mendelian randomization of 55,114 AF cases and nearly half a million referents, supporting evidence for a causal pituitary–thyroid–cardiac axis was found. Low thyrotropin and an increased ratio of triiodothyronine: free thyroxine was genetically associated with AF. However, due to the lack of genetic instruments, especially for triiodothyronine, and the lack of association with increased thyroxine, a link between a specific agent of the thyroid and AF can still not be addressed (100). Although Mendelian randomization seems a good tool for identifying causality, knowledge gaps and relatively small sample sizes can still prevent the discovery of true associations between AF risk factors and AF.

## AF Risk Prediction

### Genetic Risk Interacts With Lifestyle and Mediates Lifetime AF Risk

The incidence and prevalence of AF have increased over the past decades (101, 102), which may have influenced the increase in lifetime risk that we see in AF (37% among individuals aged >55 years) (103, 104), compared with prior estimates (men 23.8%, women 22.2% at age 55 years in the Rotterdam study; men 25.9%, women 23.2% at age 50 years in the Framingham Heart Study) (105, 106). The burden of clinical risk factors for AF contributes to the increase in lifetime risk. Advanced age is a well-known risk factor for AF, in addition to male sex, hypertension, obesity, smoking, alcohol intake, sleep apnea, diabetes, hyperthyroidism, previous myocardial infarction, and heart failure (107, 108). Clinical risk factors for AF are known to be strongly associated with arrhythmia, with large effect sizes. For instance, hypertension has been shown to increase risk of AF by 40% in women and 50% in men, diabetes increases the risk by 60% in women and 40% in men, whereas a 10-year increase in age doubles the risk for AF (109). As described above, most known genetic variants associated with AF have small effect sizes, increasing the risk for AF in the range 0.1–20.0%, except for the 4q25 locus, for which an effect size up to ~48% has been estimated (36). So how is the interplay between genetic and clinical risk factors for AF? Said et al. aimed to investigate the association of combined health behavior and genetic risk group in cardiovascular disease, including AF, in a cohort of nearly 340,000 individuals included from the UK Biobank (110). They showed that both high genetic risk and poor behavioral lifestyle are associated with increased risk of new onset of disease, yet there was no interaction effect observed between genetic risk profile and lifestyle. Participants were divided into groups according to lifestyle and genetic risk, where lifestyle was defined as ideal, poor, or intermediate, according to smoking, BMI, and physical activity. Genetic risk was calculated based on genetic variants associated with AF through GWASs. In the low genetic risk group, poor lifestyle increased the risk for new-onset AF more than 5-fold [hazard ratio (HR) 5.41, 95% CI 4.29–6.81, *P* < 0.001], compared to ideal lifestyle. Genetic composition and lifestyle had a logon additive effect on risk for disease, and the relative effect of poor lifestyle was comparable between genetic risk groups. Said et al. point to the fact that everyone benefits from lifestyle intervention, but due to the logon additive effect of genetic risk and lifestyle, high genetic risk may be a suitable selection criterium for intervention.

### Polygenic Risk Scores and Risk of Incident AF

Since common genetic variants confer small effect of risk of AF, polygenic risk scores can be constructed, summing the weighted risk of each genetic variant. Such a score can be used to evaluate the overall association of all known genetic variants with a specific phenotype. However, unlike a biomarker that represents an underlying biological pathway, the underlying biological pathways of genetic variants included in a polygenic risk score are unknown.

Lubitz et al. (111) have evaluated the association between several polygenic risk scores and incident AF. Because of the unknown true significance of each genetic variant, they used several significance thresholds and built risk scores with varying numbers (from 11 to 719) of included genetic variants. They found that polygenic risk scores were associated with AF beyond established clinical risk factors, underscoring the important contribution of genetic variants in the identification of individuals at risk for AF. The polygenic nature, the ongoing discovery of genetic variants associated with AF, and the unknown true significance of each genetic variant in the development of AF have motivated ongoing adjustments to the polygenic risk scores (111, 112). In a recent paper led by Lubitz, polygenic and clinical risk scores were combined to estimate the lifetime risk of AF in the Framingham Heart Study (104). More than 5,000 individuals were analyzed, and the overall lifetime risk of AF was estimated to be 37.1% for individuals older than 55 years. Both the polygenic risk score, including approximately a thousand genetic variants, and the clinical risk score contributed to the lifetime risk of AF. In individuals with low clinical risk for AF, the lifetime risk was doubled when moving from low to high polygenic risk [22.3% (95 CI, 15.4–29.1%) vs. 43.6% (95% CI, 35.6–51.6)], and the effect was similar in individuals with high clinical risk. Studies on polygenic risk scores for AF show that an estimation of genetic predisposition to AF is feasible with GWAS data and that polygenic risk scores can be utilized in lifetime risk models; however, regardless of the inherited predisposition to AF, the arrhythmia develops at an older age when individuals have a low burden of clinical risk factors. Modifiable risk factor management as for hypertension, obesity, smoking, and obstructive sleep apnea is still important to reduce AF risk. Future studies should determine to what extent AF risk factors can compete with polygenic AF risk.

### Polygenic AF and Risk of Stroke

Ten years ago, cardioembolic stroke was described to be associated with the two strongest AF risk loci (chromosome 4q25 and 16q22) (24, 113). In the prospective Malmö Diet and Cancer Study, incident ischemic stroke was associated with an AF genetic risk score containing 12 AF risk variants. From the 27,471 individuals, 2,160 individuals developed AF, and 1,495 developed stroke. The AF genetic risk score was associated with incident AF, but also with incident ischemic stroke. The individuals in the top AF genetic risk score quintile had a 2-fold increased risk of incident AF after adjusting for clinical risk factors, and 23% increased risk of ischemic stroke (114). Recently, in a separate analysis, Lubitz et al. described an association between increased polygenic risk for AF and stroke in both individuals with and without established AF (111). Moreover, genetic risk for AF was strongly associated with cardioembolic stroke, suggesting that an elevated polygenic risk score may serve as a surrogate for thromboembolism from AF.

The risk of stroke increases when AF is present (115, 116), but AF genetic risk was also associated with cardioembolic stroke in the absence of a diagnosis of AF. The authors hypothesize that AF genetic risk is a clinically relevant marker for subclinical or previously undiagnosed AF. If individuals with subclinical or undiagnosed AF can be identified using a polygenic risk score for AF, this may be used to prevent stroke. Thus, in the future, polygenic risk scores for AF may be clinically useful in primary and secondary prevention of AF-related stroke.

### Polygenic AF and Risk of Heart Failure

Heart failure may cause and be caused by AF. In a 38-year follow-up in the Framingham Heart study, heart failure was the strongest predictor of AF (117). Increasing age, non-ischemic etiology of heart failure, and a New York Heart Association (NYHA) class greater than II has been associated with the coexistence of AF (118). In the last 10 years, five GWAS have been conducted for heart failure. In aggregate, six genetic loci and five candidate genes were identified in 10,468 cases and 33,029 referents (119–124). Although heart failure and AF are strongly associated, only one shared genetic locus has been discovered. The ubiquitin–proteasome system enzyme 3 (*USP3*) gene is one of the novel genetic AF loci recently identified. *USP3* produces a protease that breaks down and modulates many intracellular proteins and eliminates damaged and misfolded proteins and regulates cellular processes as apoptosis. In cardiomyocytes, *USP* regulates voltage-gated membrane channels, such as sodium and potassium channels, and cell surface receptors. Cardiac signal transduction pathways are regulated by *USP* through regulating proapoptotic factors (125, 126). The onset and progression of hypertrophic cardiomyopathy are possibly linked to low concentrations or dysfunction of this protease (126, 127). The *USP*-regulated cardiac signal transduction pathways may play a mechanistic role in AF, but further studies are warranted.

The GWAS that have been reported for heart failure have had limited statistical power, compared to the half a million individuals that led to the discovery of 134 genetic variants for AF. The heterogeneity of heart failure complicates the identification of the genetic susceptibility and phenotype of heart failure. There is a large garden of variety that contributes to heart failure that can easily lead to different heart failure phenotypes. Future studies may use the polygenic risk score for AF to investigate shared genetic loci between AF and related phenotypes. The complex nature of AF may be elucidated by investigating these pleiotropic effects.

## Therapeutic Development Based on Genetic Discoveries for AF

### Rate and Rhythm Control

Data on genetic predisposition for successful rate and/or rhythm control is scarce. A study of Barret et al. investigated genetic susceptibility and rate control after intravenous treatment with diltiazem at the emergency department, using genetic variants previously associated with atrioventricular nodal conduction, resting heart rate, and AF (128). Separate risk scores were constructed for each of the three phenotypes, and the primary outcome was the maximum heart rate decrease within 4 h after diltiazem administration. The secondary outcome was a ventricular heart rate <110 bpm within 4 h of diltiazem administration, defined as rate control. However, in the 127 eligible individuals, there was no significant association between the polygenic risk score for AF and successful rate control. The relatively small sample size may render the study underpowered to reveal any significant associations.

One of the largest studies in the field was reported from Parvez et al. in the Vanderbilt AF registry, including 478 caucasian individuals with documented AF (32% women, age 63 ± 14 years), who used at least one antiarrhythmic drug. During a 12-month follow-up period, successful rhythm control (> 75% reduction in symptomatic AF burden, defined as frequency, duration and severity of symptoms, in a patient who remained on the same antiarrhythmic drug for a minimum of 6 months) was established in 399 (84%) individuals. A genetic variant at chromosome 4q25 independently predicted successful rhythm control. As described previously, the gene closest to the 4q25 locus is *PITX2*, and loss of the cardiac isoform pitx2c can lead to failure to suppress a default pathway for the sinus node development of the pulmonary myocardium or the cardiomyocytes in the left atrium (129). More studies at the intersection with genetic data and detailed information of AF treatment are needed before genetically tailored treatment will be part of the guidelines.

The predictive ability of the three most strongly associated AF susceptibility loci in relation to AF recurrence after successful electrical cardioversion has been evaluated in a cohort of 184 individuals in the Vanderbilt AF registry. One hundred sixty-two individuals underwent successful electrical cardioverson, of which 108 individuals had AF recurrence. A genetic variant at chromosome 4q25 (rs2200733) was significantly associated with AF recurrence after electrical cardioversion (130). The outcome of electrical cardioversion of AF may be genetically predisposed; however, more and well-powered studies are needed to establish true associations. Furthermore, the biological pathways of established associations should be investigated to elucidate the mechanisms of AF.

### Cardiac Catheter Ablation

Another treatment option for AF is radiofrequency catheter ablation. The use of genetic variants to predict AF catheter ablation outcome may be a promising clinical tool for the selection of patients for this procedure in the future. In 2013, Shoemaker et al. investigated 311 individuals in the Vanderbilt AF registry, of which 238 underwent only one ablation and 73 underwent two or more (131). Recurrence of AF was defined as AF, atrial flutter, or atrial tachycardia lasting more than 30 s and occuring in the blanking period of 3 months postablation. Of the 378 catheter-based ablations, 200 (53%) AF/atrial flutter recurrences were observed. The AF risk allele, rs2200733, at the 4q25 locus predicted shorter recurrence-free time after AF ablation.

In 2015, Shoemaker et al. explored the three genetic loci related to *PITX2, KCNN3*, and *ZFHX3* that have been consistently been strongly associated with AF (132). In a combined cohort of Vanderbilt University, Heart Center Leipzig, and Massachusetts General hospital, 991 individuals with first-time AF ablation were analyzed. AF recurrence rate, defined as AF, atrial flutter, or atrial tachycardia lasting more than 30 s and occuring after 3 months postablation, was 42% during 12 months of follow-up. However, only the genetic variant rs2200733 at 4q25 predicted increased risk of recurrence.

Park et al. performed several analyses in the Korean Yonsei AF ablation population. They found that the genetic variant rs2106216 at the *ZFHX3* locus on chromosome 16q22 was independently associated with a good response after AF ablation, but no association was found with the *PITX* locus. So far, the findings of Park et al. have not been replicated. The difference in ethnicity, patient characteristics, and the consistenty in ablation technique and relatively small sample size may explain the different results with previous studies (131–133). Recently, a polygenic risk score for AF was constructed with four genetic variants, rs1448818, rs2200733, rs6843082, and rs6838973, from the *PITX2* locus and one from the *ZFHX3* locus, rs2106261, to predict AF recurrence after ablation in another Korean population (134) from the Seoul National University Hospital and Korea University Guro Hospital. During 23 months of follow-up in 746 individuals (74% men, mean age = 59 ± 11 years), 168 (22.5%) had AF recurrence after AF catheter ablation. Increased polygenic risk for AF was strongly associated with AF recurrence; Individuals carrying 7 to 10 risk alleles of the 5 AF risk variants (rs1448818, rs2200733, rs6843082, rs6838973, and rs2106261) had a 2.66-fold increased risk of recurrence compared to the lowest risk group, where individuals carried zero to three risk alleles.

Large, well-phenotyped populations are necessary to discover new genetic variants associated with outcomes of AF ablation, e.g., a large-scale AF ablation GWAS may expose more common genetic variants related to AF recurrence after ablation. However, even when associations of genetic variants with AF ablation are established, underlying biological pathways leading to AF recurrence after ablation are still unknown. In aggregate, the current literature does not support the use of polygenic risk scores to guide AF management.

## Patient Specific Management—Where Do We Go From Here?

**Figure 3:**
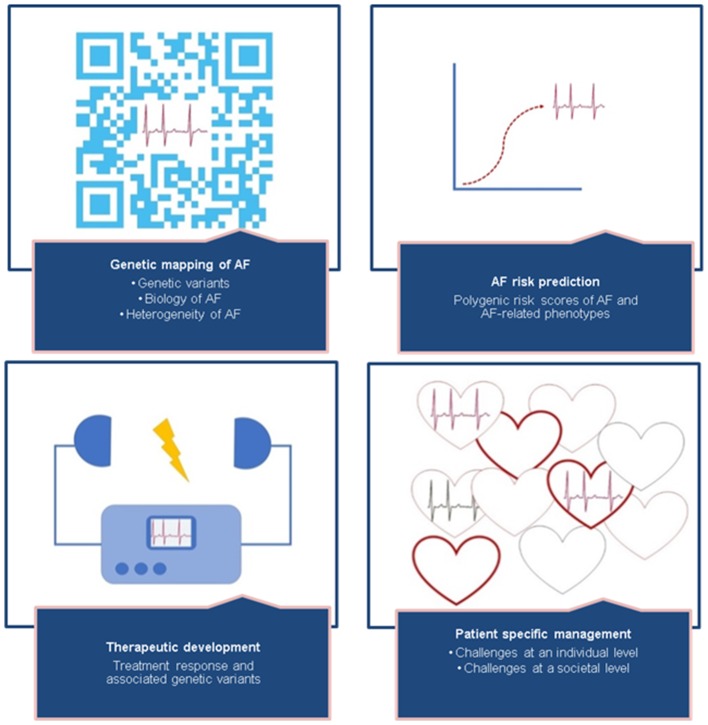
The missing heritability of AF can be revealed by focusing on mapping the polygenic structure of AF, improving risk prediction, therapeutic development, and patient-specific management. Details of suggested studies are described in Table 3. Future perspectives—translating AF genetics into clinical practice.

The complex biology of AF and AF heritability has been increasingly elucidated in the era of AF genetics research. Nevertheless, our knowledge of the biologic mechanisms underlying the genetic associations is still limited. The translation of genetic information into clinical practice is difficult and far from ready to be implemented. So where do we go from here (Figure 3)?

First, we have to unravel the missing heritability of AF. Although *genetic mapping of AF* is still ongoing, the discovery of novel genetic variants has made great leaps in the past decade, revealing 134 common genetic variants for AF. Undiscovered common genetic variants are still part of the missing heritability, and therefore, the search in AF GWAS studies should be continued. Efforts are also underway to pursue larger whole-genome sequencing studies, which may identify common and rare genetic variants for AF. Another important avenue is for more studies to focus on functional characterization of genetic variants, through expression quantitative trait loci (eQTL) analyses, proteomic, transcriptomic, metabolomic, cell and animal experiments, as well as further large-scale population-based studies revealing gene–gene and gene–environment interactions will expand the knowledge of the biology underlying AF.

We need to better understand the mechanisms underlying the complex pleiotropic relationship between AF and AF-related phenotypes. AF is known to be associated with multiple clinical risk factors, which may interact with or be the cause of AF. Well-powered Mendelian randomization analyses of AF and AF-related phenotypes, including endophenotypes such as ECG markers, may elucidate causal relations. Phenome-wide association studies (PheWAS) can be performed to identify associations between a genetic variant and multiple phenotypes, revealing novel genotype–phenotype associations.

Similarly to clinical *AF risk prediction* models, such as the CHARGE-AF risk score (135), we anticipate that polygenic risk scores for AF will be used in daily clinical practice in the future to guide prevention and treatment of AF. Polygenic risk scores may be used to reveal AF, but also AF-related phenotypes, like stroke, may attribute to AF mechanisms. Polygenic risk scores are a hot topic and have been increasingly performed in several fields of medical research. It is essential to view the clinical applicability of polygenic risk scores in perspective; unlike other clinical biomarkers, the underlying biological pathways of the genetic variants combined in a risk score are still largely unknown. There has been a high hope that discovering genetic causes of AF would also lead to the *development of new drugs* to treat the arrhythmia. It has been suggested that selecting drug targets that have a known genetic association with its related phenotype doubles the likelihood of success in developing new drugs (136). Although in the past decades the number of studies of genetic variants for AF increased extensively, underlying biological pathways are still unclear. The process has so far been challenging when it comes to development of new drugs for AF, mainly due to the fact that many novel genetic loci for AF are not directly “druggable” but require years in the wet-lab for their functional effects to be mapped out. Identifying statistical associations between genetic variants and AF without knowing neither (I) which specific parts of the genetic loci carry the causal effects nor (II) the underlying biological mechanisms carried by the causal variants will not automatically increase our success in establishing new therapeutic targets. Although there are a few new and promising drugs emerging that have been developed partly due to supporting genetic evidence (39–42), the yield of genetically identified drug targets has been disappointing. We need to identify the causal entities in the AF loci identified and disentangle their biological mechanisms in order to improve the development of new drugs and to understand how to improve AF interventions.

The complex and heterogeneous nature of AF demands *patient-specific management*. The current clinical classification of AF is not optimal, as it basically relies on the frequency and duration of AF episodes, and not underlying disease mechanisms, comorbidity, or daily function. Hence, the classification does not reflect the phenotypic and genotypic spectrum of AF, nor the fundamental difference between monogenic and polygenic AF forms. In association with disease, rare genetic variants tend to have a large effect and may represent monogenic disease, which has shown to be the case in several reports of familial AF (137). In families with known monogenic causes of AF, disease risk may be more easily predictable, depending on the penetrance, and it will potentially be possible to directly target the single gene or biological pathway involved in disease development. Nevertheless, individually targeted treatment of monogenic AF may be challenging due to the heterogenic nature of monogenic AF—almost no family carries the same genetic variant as the cause of their disease. Historically, monogenic variants leading to familial AF are involved in electrical abnormality. However, as we have discussed, more recent studies of familial AF have also revealed variants related to structural changes. In contrast, more common AF phenotypes are more likely to be multifactorial and polygenic, with multiple common genetic variants, as well as environmental factors, contributing to complex disease development, thus challenging both targeted treatment and estimation of disease risk. Although monogenic AF represents only a minority of AF cases, genetic information derived from this group provides valuable knowledge for interpreting data derived from GWAS and high-throughput sequencing and in the search for targeted treatment for more common forms of AF. Through increased insight into genetic causality and the various biological pathways implicated, it will open up for better classification of AF, thereby providing better opportunities for risk assessment and precision medicine for all types of AF. We have described a range of studies highlighting the possibility of individualized risk assessment, based on both genetic and clinical risk, and implicated the importance of addressing modifiable clinical risk factors, such as hypertension and obesity to prevent or delay the onset of AF. Potential challenges in clinical practice will be how to convey the concept of genetic risk to the individual patient and tackling the potential reverse effect on behavior when a patient gets insight into her or his own genetic risk. Another challenge can be the cost of implementing genetic screening of AF globally. However, potential reductions in the high lifetime risk of AF and costs of only partially effective treatments may contribute to a positive cost–benefit ratio of the introduction of genetic screening for AF. Overall, better understanding of the genetics of AF will hopefully provide better tools to prevent, detect, and treat AF, thus reducing the impact of the disease both at an individual level and in a public healthcare perspective (Table 3).

**Table 3:** Knowledge gaps in the genetics of AF.

**Direction**	**Knowledge gaps**	**Suggested studies**
Genetic mapping of AF	*Total number of genetic variants*	•GWAS: Larger, non-European ancestry groups, more specific AF phenotypes •Whole-exome and -genome sequencing
	*Biology of AF*	• Expand eQTL studies • Network analyses • *In vivo* and *in vitro* experiments • Gene–gene interaction • Epigenetics • Transcriptomics • Proteomics • Metabolomics
	*Heterogeneity of AF*	• Discover new AF clinical risk factors • Validate AF risk models • Genetic classification of AF phenotypes • Investigate gene–environment interaction • PheWAS • Mendelian randomization studies of AF risk factors
AF prediction	*Polygenic risk scores*	• Improve genetic risk scores for AF, AF subtypes, and AF-related phenotypes
Therapeutic development	*Treatment response*	• Improve genetic risk scores for AF treatment • Increase cohorts with both genetics and treatment response data • Investigate the underlying biological mechanisms of genetic variants to identify new drug targets
Patient specific management	*Individual concerns*	• Focus on individualized risk assessment • How to relate to AF genetics and the ethical concept and resulting patient behavior
	*Societal concerns*	• Cost-effective consequences

## Author Contributions

All authors listed have made a substantial, direct and intellectual contribution to the work, and approved it for publication.

### Conflict of Interest Statement

The authors declare that the research was conducted in the absence of any commercial or financial relationships that could be construed as a potential conflict of interest.
